# Decreased Expression of the Aryl Hydrocarbon Receptor in Ocular Behcet's Disease

**DOI:** 10.1155/2014/195094

**Published:** 2014-06-22

**Authors:** Chaokui Wang, Zi Ye, Aize Kijlstra, Yan Zhou, Peizeng Yang

**Affiliations:** ^1^The First Affiliated Hospital of Chongqing Medical University, Chongqing Key Lab of Ophthalmology, Chongqing Eye Institute, Youyi Road 1, Chongqing 400016, China; ^2^University Eye Clinic Maastricht, Maastricht, The Netherlands

## Abstract

Recent studies show that the aryl hydrocarbon receptor (AhR) is involved in immune responses. AhR is activated following interaction with its ligands, such as 6-formylindolo[3,2-b]carbazole (FICZ) and 2-(1′H-indole-3′-carbonyl)-thiazole-4-carboxylic acid methyl ester (ITE). In this study, we investigated the role of AhR activation by its endogenous ligands in the pathogenesis of ocular Behcet's disease (BD). The expression of AhR was significantly decreased in active BD patients as compared to inactive BD patients and normal controls. Both FICZ and ITE inhibited Th1 and Th17 polarization and induced the expression of IL-22 by PBMCs and by CD4^+^T cells in active BD patients and normal controls. Stimulation of purified CD4^+^T cells with FICZ or ITE caused a decreased expression of RORC, IL-17, IL-23R, and CCR6 and an increased phosphorylation of STAT3 and STAT5. The present study suggests that a decreased AhR expression is associated with disease activity in BD patients. The activation of AhR by either FICZ or ITE was able to inhibit Th1 and Th17 cell polarization. Further studies are needed to investigate whether modulation of AhR might be used in the treatment of BD.

## 1. Introduction

Behcet's disease (BD) is a chronic systemic inflammatory disease affecting the eye, skin, oral mucosa, gastrointestinal tract, and central nervous system [1]. It is a relatively common uveitis entity in China and the clinical ocular features have been described extensively elsewhere [2]. Although the pathogenesis of BD is still not completely understood, it is currently thought that environmental factors may trigger the development and recurrence of this disease in a genetically susceptible host [3]. It is classified as an example of an autoinflammatory disorder with marked involvement of both Th1 and Th17 lymphocyte subsets [4–7]. Consistently, strategies aimed at suppressing the abnormal Th1 and Th17 cell response have been reported as a therapeutic approach in BD patients, which is supported by findings in experimental autoimmune uveitis (EAU) models in mice [8, 9].

The Aryl hydrocarbon receptor (AhR) is ubiquitously expressed in vertebrate cells and is well known to mediate toxic effects of several environmental pollutants, including polycyclic- and halogenated aromatic hydrocarbons such as benzo(a)pyrene (B(a)P) and 2,3,7,8 tetrachlorodibenzo-p-dioxin (TCDD) [10]. Other ligands include natural dietary substances, heme metabolites, and tryptophan photoproducts [11, 12]. AhR is a ubiquitous transcription factor present in the cytoplasm, which, after binding to its ligands, translocates into the nucleus where it attaches to its dimerization partner AhR nuclear translocator. The AhR/AhR nuclear translocator complex initiates transcription of a variety of genes with promoters containing a so-called dioxin-responsive element consensus sequence, which ultimately results in a variety of toxic or biochemical responses [10]. In addition to its role in mediating toxic responses, the AhR pathway has many other physiological roles. Mouse strains deficient in the AhR protein showed defects in neuronal development and photoreceptor development, decreased animal weights, fatty metamorphosis and portal tract fibrosis in the liver, and poor fecundity [13, 14]. It has also been reported that AhR plays an important role in vascular development. The most overt phenotype of the AHR knockout mouse is a markedly reduced liver size, which is owing to defects in the resolution of fetal vascular structure. AhR-null mice also showed abnormalities in the vascular architectures of the kidney, liver sinusoids, and eye, including persistence of the embryonic hyaloid artery. In adults, the knockout of AhR is linked to cardiac hypertrophy, hypertension, and elevated levels of the potent vasoconstrictors endothelin-1 and angiotensin II [14]. Taken together, AhR plays a critical role in the normal physiological function.

More recent studies have shown that the AhR also plays a critical role in the immune response. AhR-deficient mice, for instance, develop a more severe form of experimental autoimmune encephalomyelitis (EAE) as compared to wild-type mice [15, 16]. Studies in mice also showed that AhR activation can alter the differentiation of Treg cells and Th17 cells in a ligand-specific manner [15]. AhR activation by TCDD was able to suppress EAU and EAE by inducing CD4^+^CD25^+^Foxp3 Treg cell differentiation. On the other hand, AHR activation by the ligand 6-formylindolo[3,2-b]carbazole (FICZ) aggravated the severity of EAE by inhibiting the development of Treg cells and promoting the differentiation of Th17 cells [15]. Although TCDD may block the autoimmune response, it also causes various toxic responses, including cellular damage and carcinogenesis [17]. Treating EAE mice with another AHR ligand, 2-(1′H-indole-3′-carbonyl)-thiazole-4-carboxylic acid methyl ester (ITE), which is an endogenous nontoxic tryptophan-derived AhR ligand, resulted in a significant reduction of the inflammatory response, increased FoxP3^+^Treg cells, and resulted in an enhanced production of tolerogenic dendritic cells [18]. In the animal models of collagen-induced arthritis (CIA), it was shown that AhR deficiency ameliorated the severity of CIA by inhibiting the proinflammatory cytokines as IL-1*β* and IL-6 as well as Th17 cell response [19]. In human studies, AhR activation by FICZ or TCDD in CD4^+^T cells was shown to inhibit the production of IL-17A while simultaneously promoting the production of the immunoprotective cytokine IL-22 [20, 21]. Taken together these data indicate that, depending on the cell type analyzed and the AhR ligands used, AhR activation can differentially modulate the Th cell response and act as initiator or attenuator of T cell-driven autoinflammatory responses.

Given the deregulated Th1 and Th17 cell response in BD patients, we investigated whether AhR signaling could be exploited to inhibit the development of T cell responses in BD patients. Here we found that the gene expression of AhR was decreased in active BD patients. AhR activation by either FICZ or ITE inhibited the Th1 and Th17 cell polarization and induced IL-22 production by PBMCs and CD4^+^T cells. The effect of FICZ and ITE on CD4^+^T cells was associated with a decreased expression of RORC, IL-17, IL-23R, and CCR6 and an increased phosphorylation of STAT3 and STAT5.

## 2. Materials and Methods

### 2.1. Subjects

Thirty active BD patients (18 men and 12 women, with an average age of 34.3 years) and nineteen inactive BD patients (11 men and 8 women, with an average age of 36 years) were included in this study. Forty-nine age- and gender-matched healthy volunteers were included as normal controls. The criteria of the International Study Group for BD were used to diagnose BD [22]. Active intraocular inflammation was defined by the presence of nongranulomatous keratic precipitates (100%), flare and cells in the anterior chamber (100%), vitreous cells (75%), and retinal vasculitis (100%) as shown by fundus fluorescein angiography (FFA). The extraocular manifestations were recurrent oral aphthous ulcers (100%), multiform skin lesions (80%), arthritis (36%), and recurrent genital ulcers (30%). The most frequent skin symptom in the patients included in our study was erythema nodosum, and the most frequent combination of skin lesions was erythema nodosum with papulopustular lesions. Active BD patients included in our study refer to the patients with Behcet's disease when visiting us all showed active intraocular inflammation; meanwhile, some of them had active extraocular findings, such as oral ulcers, genital ulcers, or skin lesions and some had a previous history of extraocular findings. The active BD patients enrolled in this study were all on their first visit to our hospital. These patients did not use any immunosuppressive agents or have received a low dose of immunosuppressive agents or prednisone but have stopped at least 2 weeks prior to blood sampling. We normally treated these active BD patients using systemic corticosteroids in combination with cyclosporine, cyclophosphamide, or chlorambucil for more than one and a half years. The drug dose was gradually tapered after the intraocular inflammation and other extraocular findings were controlled and the treatment usually stopped 6 months after complete control of the intraocular inflammation. After termination of all medications for at least 2 months we collected the blood sample from those inactive BD patients. The inactive patients were those who did not have any active inflammation in the eye as well as extraocular organs following a long term treatment with systemic corticosteroids combined with other immunosuppressive agents. We collected the peripheral blood sample of the outpatients with Behcet's disease and corresponding controls mostly in the afternoon. Written and informed consent was obtained from all patients and normal controls. All procedures followed the tenets of the Declaration of Helsinki and were approved by the Clinical Ethical Research Committee of Chongqing Medical University.

### 2.2. Cell Culture

Peripheral blood samples were obtained from BD patients and healthy volunteers. Preparation of PBMCs and CD4^+^T cells was performed as described earlier [23]. Human CD4^+^T cells were isolated from PBMCs by human CD4 microbeads (purity > 90%; Miltenyi Biotec, Palo Alto, CA) according to the manufacturer's instructions. The PBMCs and purified CD4^+^T cells were cultured at 1 × 10^6^ in 24-well plates in RPMI1640 medium supplemented with 10% FBS. A combination of anti-CD3 and anti-CD28 antibodies (2 ug/mL) (eBioscience, San Diego, Calif) was used to activate PBMCs for 3 days, and a combination of anti-CD3/CD28 (2 ug/mL) and IL-23 was used to activate CD4^+^T cells for 6 days. When used, 0.05% DMSO (control), FICZ (100 nmol/L) (Enzo Life Sciences, USA), and ITE (100 nmol/L, Tocris Bioscience, USA) were added at the beginning of the culture. The supernatants were collected for cytokine measurement by ELISA and the cells were harvested and used for FACS analysis or mRNA quantification.

### 2.3. Flow Cytometry

AnnexinV-FITC/PI Kit (KeyGen Biotechnology, Nanjing, China) was used to evaluate the effect of FICZ and ITE on the apoptosis of PBMCs. For analysis of the frequency of Th1 and Th17, the cells were stimulated by adding PMA (50 ng/mL, Sigma-Aldrich, St. Louis, MO) and ionomycin (1 ug/mL, Sigma) for 1 h at 37°C. Then, brefeldin A (10 ug/mL, Sigma) was added for another 4 h; the cells were fixed and permeabilized using the eBioscience Cytofix/Cytoperm kit according to the manufacturer's instructions and then incubated with CD3-(PerCP)-Cy5.5, anti-human CD8-APC, anti-human IL-17A-PE, and anti-human IFN-*γ*-FITC (BD Biosciences). For phosphorylated STAT staining, stimulated CD4^+^T cells were fixed with Fix buffer (BD Biosciences) for 10 min at 37°C, permeabilized with Perm buffer (BD Biosciences) for 30 min on ice, and stained with anti-human pSTAT3-PE, anti-human pSTAT4-Per-cy5.5, anti-human pSTAT4-Per-cy5.5, anti-human pSTAT5-PE, or isotype control mAbs (BD Biosciences). Flow cytometric analysis was performed on a FACScan flow cytometer (BD Biosciences) to measure mean fluorescence intensity (MFI). Results were expressed as the percentage difference compared with isotypic control (IC) using the formula [mean fluorescence intensity (MFI) of sample − MFI of IC]/MFI of IC.

### 2.4. Quantitative RT-PCR

To detect the expression of AhR mRNA in BD patients and normal controls, total RNA was extracted from PBMCs of active BD patients, inactive BD patients, and normal controls by using a commercially available kit (RNeasyPlus Mini kit; Qiagen, Valencia, California) according to the manufacturer's instructions. To investigate the effect of AhR activation by FICZ or ITE on the expression of associated molecules of Th1 and Th17 cells, isolated CD4^+^T cells (purity > 90%) were stimulated with antiCD3/CD28 and IL-23 with or without AhR ligands for 6 days; then, the cells were harvested for T-bet, RORC, IL-17, CCR6, and IL-23R mRNA quantification. Total RNA was isolated from stimulated CD4^+^T cells using a commercially available kit (RNeasyPlus Mini kit; Qiagen, Valencia, California). Reverse transcription of RNA of the PBMCs or stimulated CD4^+^T cells was performed using the Superscript III Reverse Transcriptase system (Invitrogen, Carlsbad, CA, USA). Amplification of transcripts was performed using SYBR RT-PCR (Takara, Dalian, China) and run on AB 7500 Fast System (Applied Biosystems). The following primers were used for real-time PCR: T-bet forward: 5′-GATGCTGCCAGGAAGTTTCAT-3′ and reverse: 5′-GCACAATCATCTGGGTCACATT-3′ and *β*-actin forward: 5′-GGATGCAGAAGGAGATCACTG-3′ and reverse: 5′-CGATCCACACGGAGTACTTG-3′. The primer sequences of IL-23R, IL-17, RORC2, and CCR6 were used as described elsewhere [19]. For AhR, Quantitect Primers (Qiagen, Valencia, CA) were used. The expression of each gene was normalized to the expression of *β*-actin using the 2^−ΔΔCT^ method as described previously [24].

### 2.5. ELISA

IL-17, IFN-*γ*, and IL-22 levels in the cell culture supernatants were measured with ELISA kits (R&D Systems, Minneapolis, MN) according to the manufacturer's protocols.

### 2.6. Statistical Analysis

The statistical significance of differences was determined by the Kruskal-Wallis test and Mann-Whitney test, Independent-Sample* t* test, Paired-sample* t* test, or Wilcoxon's matched-pairs test. *P* < 0.05 was considered statistically significant. All analyses were performed using commercially available statistical software (SPSS 12.0; SPSS Inc., Chicago, Illinois).

## 3. Results

### 3.1. Decreased AhR mRNA Expression in PBMCs from Active BD Patients

**Figure 1:**
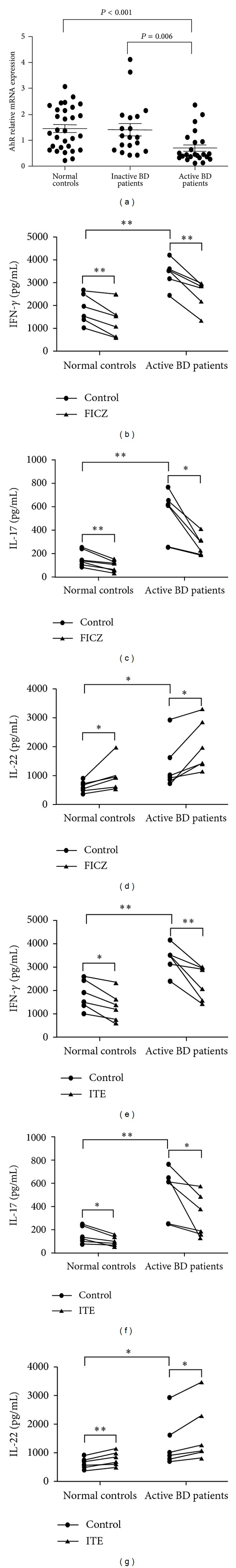
AhR mRNA expression is decreased in active BD patients and AhR activation by FICZ and ITE inhibits IFN-*γ* and IL-17 and induces IL-22 production by PBMCs. (a) AhR mRNA was evaluated in PBMCs from active BD patients (*n* = 23), inactive BD patients (*n* = 19), and normal controls (*n* = 30) by real-time PCR and normalized to *β*-actin. The Kruskal-Wallis test and Mann-Whitney test were used to analyze the statistical difference between the various groups. Data are expressed as mean ± s.e.m. (b–g) PBMCs from active BD patients (*n* = 6) and normal controls (*n* = 6) were stimulated with anti-CD3/CD28 in the presence or absence of FICZ (100 nmol/L) or ITE (100 nmol/L) for 3 days. The supernatants were harvested for detection of IFN-*γ* (b and e), IL-17 (c and f), and IL-22 (d and g) by ELISA. Paired-sample* t* test or Wilcoxon's matched-pairs test for related samples and Independent-Sample* t* test for independent samples were used for statistical analyses. *P < 0.05, **P < 0.01.

PBMCs from active BD patients, inactive BD patients, and normal controls were used to assay the mRNA expression of AhR. The results showed that AhR mRNA expression was significantly decreased in active BD patients as compared to inactive BD patients (*P* = 0.006) and normal controls (*P* < 0.001). There was no significant difference concerning AhR mRNA expression between inactive BD patients and normal controls (Figure 1(a)).

### 3.2. FICZ and ITE Inhibit Th1 and Th17 Cell Polarization and Induce IL-22 Expression by PBMCs from BD Patients and Normal Controls

**Figure 2:**
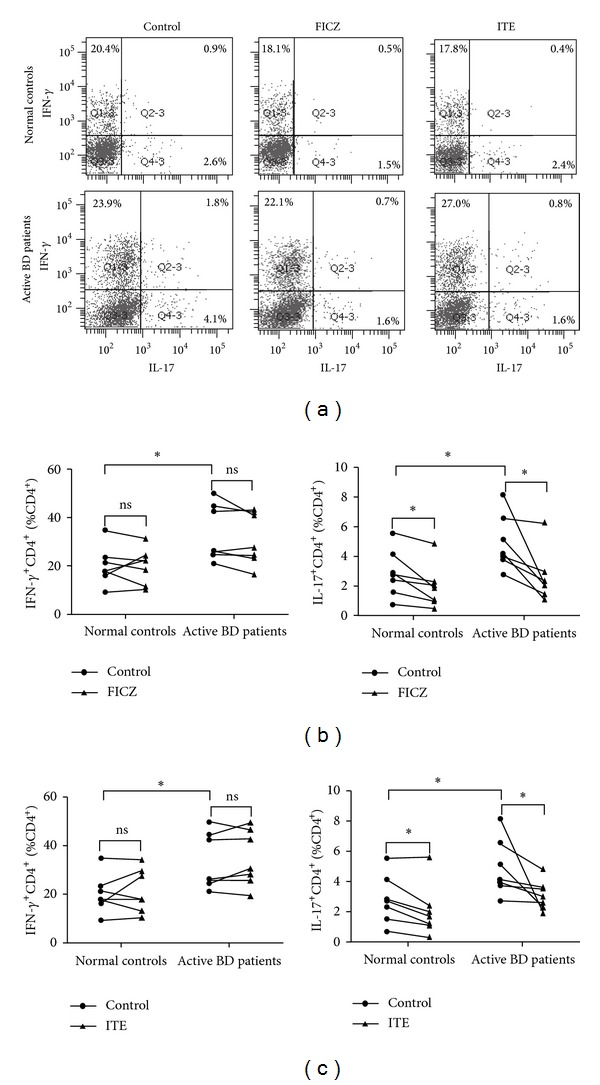
FICZ and ITE inhibit the frequency of IL-17-expressing CD4^+^T cells but have no effect on IFN-*γ*-expressing CD4^+^T cells in PBMCs. PBMCs from active BD patients (*n* = 7) and normal controls (*n* = 7) were stimulated with anti-CD3/CD28 in the presence or absence of FICZ (100 nmol/L) or ITE (100 nmol/L) for 3 days. The cells were analyzed for intracellular expression of IFN-*γ* and IL-17 by flow cytometry. (a) Dot plots of a representative subject for each group are shown. (b and c) Quantitative analysis of the percentage of IFN-*γ*- and IL-17-expressing CD4^+^T cells. Paired-sample* t* test or Wilcoxon's matched-pairs test for related samples and Independent-Sample* t* test for independent samples were used for statistical analyses. *P < 0.05, ns: not statistically different.

Because an increased frequency of Th1 and Th17 cells has been shown to be associated with the inflammatory activity of BD and since AhR has been reported to be involved in T cell immune responses, we next determined the effect of endogenous AhR ligands on the Th1 and Th17 cell response in active BD patients and normal controls. PBMCs were stimulated with anti-CD3/CD28 to mimic antigen presentation in the presence or absence of FICZ or ITE. The apoptotic effect of FICZ and ITE was first evaluated by flow cytometry. Annexin V and PI double staining showed that FICZ and ITE had no significant influence on the apoptosis of PBMCs (Supplementary Figure 1, available online at http://dx.doi.org/10.1155/2014/195094). IFN-*γ*, IL-17, and IL-22 production in cell culture supernatants by these stimulated PBMCs from active BD patients were higher as compared to normal controls. Addition of FICZ or ITE significantly inhibited the production of IFN-*γ* and IL-17 but enhanced IL-22 production in both the BD and the control groups (Figures 1(b)–1(g)). Flow cytometry analysis showed an increased frequency of IL-17 and IFN-*γ*-expressing CD4^+^T cells in PBMCs obtained from active BD patients as compared to normal controls. FICZ and ITE significantly inhibited the frequency of IL-17-expressing CD4^+^T cells in both BD patients and in the controls, whereas no detectable effect was observed on the percentage of IFN-*γ*-expressing CD4^+^T cells (Figure 2).

### 3.3. FICZ and ITE Directly Inhibit Th1 and Th17 Cell Polarization and Induce IL-22 Expression by CD4^+^T Cells from BD Patients and Normal Controls

**Figure 3:**
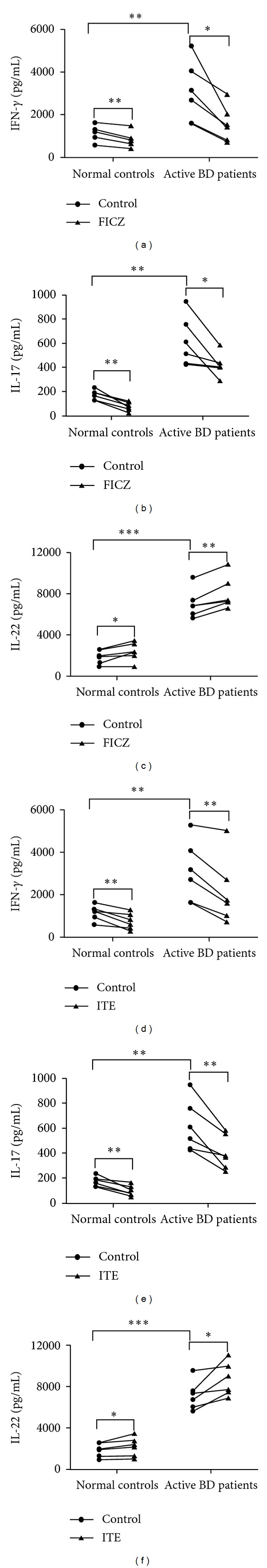
FICZ and ITE inhibit IFN-*γ* and IL-17 but induce IL-22 expression by CD4^+^T cells from BD patients and normal controls. Purified CD4^+^T cells from active BD patients (*n* = 6) and normal controls (*n* = 6) were stimulated with anti-CD3/CD28 and rIL-23 in the presence or absence of FICZ (100 nmol/L) or ITE (100 nmol/L) for 6 days. IFN-*γ* (a and d), IL-17 (b and e), and IL-22 (c and f) production in the supernatants were determined by ELISA. Paired-sample* t* test or Wilcoxon's matched-pairs test for related samples and Independent-Sample* t* test for independent samples were used for statistical analyses. *P < 0.05, **P < 0.01, and ***P < 0.001.

**Figure 4:**
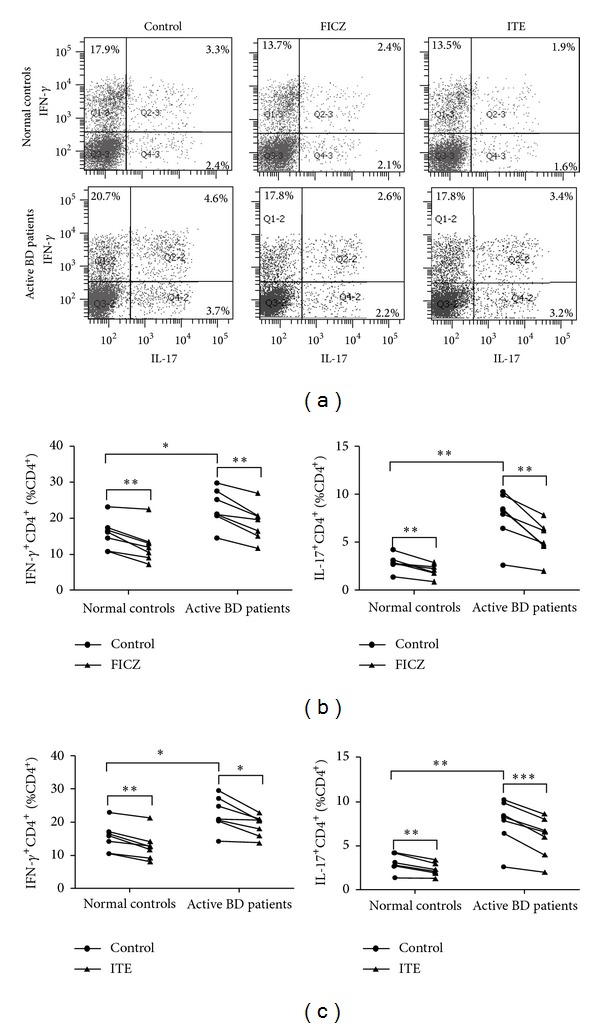
FICZ and ITE inhibit Th1 and Th17 cell polarization by CD4^+^T cells from BD patients and normal controls. Purified CD4^+^T cells from active BD patients (*n* = 7) and normal controls (*n* = 7) were stimulated with anti-CD3/CD28 and rIL-23 in the presence or absence of FICZ (100 nmol/L) or ITE (100 nmol/L) for 6 days. The cells were collected and analyzed for intracellular expression of IFN-*γ* and IL-17 by flow cytometry. (a) Dot plots of a representative subject for each group are shown. (b and c) Quantitative analysis of the percentage of IFN-*γ*- and IL-17-expressing CD4^+^T cells. Paired-sample* t* test or Wilcoxon's matched-pairs test for related samples and Independent-Sample* t* test for independent samples were used for statistical analyses. *P < 0.05, **P < 0.01, and ***P < 0.001.

The aforementioned results showed that FICZ and ITE can inhibit Th1 and Th17 cell polarization and induced the expression of IL-22 by PBMCs. As CD4^+^T cells play a critical role in the pathogenesis of Behcet's disease [4], we next investigated whether FICZ and ITE were able to influence the development of Th1 and Th17 cells. The results showed that stimulation of purified CD4^+^T cells from active BD patients resulted in a higher level of IFN-*γ*, IL-17, and IL-22 production in the cell culture supernatants as compared to normal controls. The addition of FICZ and ITE significantly inhibited the production of IFN-*γ* and IL-17 but induced IL-22 production by stimulated CD4^+^T cells (Figure 3). Consistent with the ELISA, intracellular cytokine analysis by flow cytometry revealed a significantly increased percentage of IFN-*γ*- and IL-17-expressing CD4^+^T cells in active BD patients as compared to normal controls. AhR activation by either FICZ or ITE inhibited the frequency of IFN-*γ*- and IL-17-expressing CD4^+^T cells in active BD patients and normal controls. No detectable difference was observed concerning the inhibitory effect between the two tested ligands (Figure 4).

**Figure 5:**
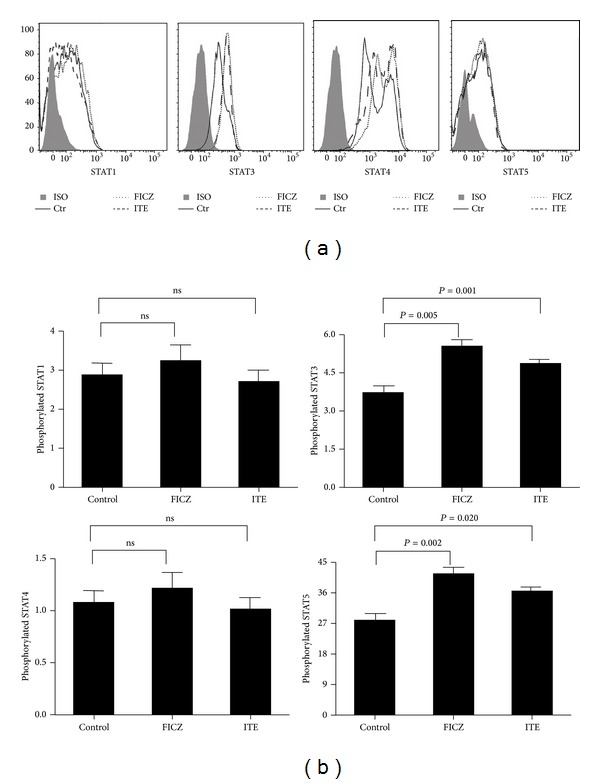
Effect of AhR activation by FICZ or ITE on phospho-STAT1, -3, -4, and -5 in CD4^+^T cells. (a) CD4^+^T cells from normal controls (*n* = 7) were stimulated with anti-CD3/CD28 and rIL-23 in the presence or absence of FICZ or ITE for 30 min. Intracellular phosphorylated STAT1, -3, -4, and -5 were analyzed by flow cytometry. A representative histogram from each group along with the isotype control is shown. (b) Statistical results of phosphorylated STAT1, -3, -4, and -5 in stimulated CD4^+^T cells. Paired-sample* t* test for related samples was used for statistical analyses. The data are expressed as mean ± s.e.m.

To investigate the mechanism by which AhR signaling in CD4^+^T cells might modulate the expression of IFN-*γ*, IL-17, and IL-22, we assessed the signaling activity of STAT pathways, which are critically involved in the differentiation of different Th cell subsets. The results showed that AhR activation by FICZ or ITE in CD4^+^T cells cultured in the presence of antiCD3/CD28 and rIL-23 induced STAT3 and STAT5 phosphorylation. However, phosphor-STAT1 and phosphor-STAT4 were only detected at low levels and we could not detect an effect of AhR activation by FICZ or ITE on STAT1 and STAT4 phosphorylation (Figure 5).

**Figure 6:**
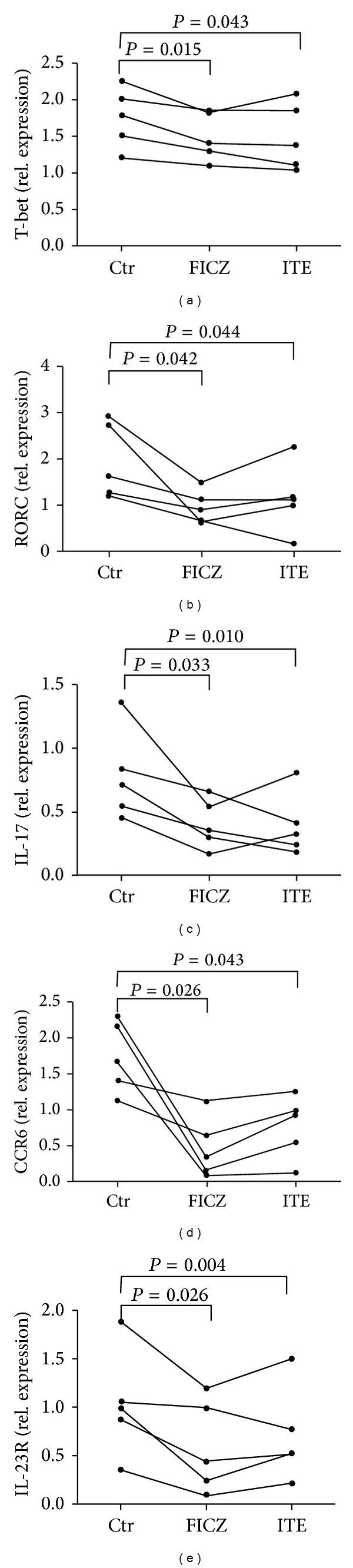
FICZ and ITE inhibit the molecules associated with Th1 and Th17 cell effector function in stimulated CD4^+^T cells. CD4^+^T cells from healthy controls (*n* = 5) were cultured with anti-CD3/CD28 and rIL-23 in the presence or absence of FICZ or ITE for 6 days. The cells were harvested for mRNA analysis of T-bet, RORC, IL-17, IL-23R, and CCR6 expression by real-time PCR. Paired-sample* t* test for related samples was used for statistical analyses.

As polarization of CD4^+^T cell subsets is regulated by transcription factors [25], we next investigated whether FICZ or ITE had an influence on the transcription factors of Th1 and Th17 cells. We found that AhR activation by FICZ or ITE significantly inhibited the expression of T-bet and RORC mRNA in CD4^+^T cells. Furthermore, we observed that FICZ or ITE not only inhibited the gene expression of IL-17 but also suppressed the gene expressions of IL-23R and CCR6 which are essential to both the maintenance and function of Th17 cells, indicating that FICZ and ITE interfere with Th17 polarization and function (Figure 6).

## 4. Discussion

In this study we found a decreased gene expression of AhR in PBMCs of active BD patients as compared to inactive BD patients and normal controls. These findings suggest a role for AhR expression in the pathogenesis of BD. Most studies in the past on AhR have been dedicated to its role in mediating toxicity [10] by dioxins but it is now emerging that AhR plays a physiological role in the immune response and that it is highly expressed on dendritic cells as well as on Th17 cells [26]. The role of AhR in clinical autoimmune disease is a novel area of research and expression of AhR on PBMCs obtained from active BD patients has, to our knowledge, not yet been addressed before. Interestingly, we found a significantly decreased AhR expression in active BD patients as compared to inactive BD patients and normal controls. It is well known that some immunosuppressive agents or prednisolone has a long effect on the immune system. The decreased AhR expression in active BD patients treated by a low dose of immunosuppressive agents or prednisolone seems to exclude the influence of these immunosuppressive agents or prednisolone on AhR expression. However, it is interesting to address the influence of immunosuppressive agents or prednisolone on AhR expression; this can be achieved by our further studies. It is well established that male BD patients run a more active course as compared to female patients and that this disease may involve different organs [2]. Therefore, it is necessary to clarify whether there is difference in male and female patients as well as among those with different organ involvement using more prehensive studies in the future.

To provide further evidence for a possible role of the AhR in the pathogenesis of BD we performed a series of experiments whereby we investigated the role of two known endogenous AhR ligands on the function of T cells. Consistent with previous results from our group, we observed that PBMCs obtained from active BD patients showed an elevated production of IFN-*γ*, IL-17, and IL-22 as compared to normal controls [4, 27]. Addition of the AhR ligands FICZ and ITE to these PBMC cultures resulted in a significant inhibition of the production of IFN-*γ* and IL-17 and an increased expression of IL-22. Because CD4^+^T cells play a critical role in the development of autoimmune diseases [25], including BD [4], we further tested whether there was a direct effect of FICZ and ITE on cytokine production using purified CD4^+^T cells. CD4^+^T cells showed the same result as obtained earlier with PBMCs. Intracellular staining confirmed the above results except that the ligands did not affect the frequency of IFN-*γ*-producing CD4^+^T cells from PBMCs. The reasons for the discrepancy between intracellular and extracellular IFN-*γ* expression is not clear and warrants further study.

Our findings with FICZ are consistent with data of recent studies which showed that AhR activation was able to induce production of IL-22 and that it could inhibit IL-17 production [20]. The findings are however in disagreement with results showing that FICZ induced IL-17 production in the mouse [28, 29]. Our findings with ITE were consistent with the in vivo inhibitory effect on the production of IFN-*γ* and IL-17 in mice [18]. All together, these data provide evidence that FICZ and ITE may act as a negative regulator for the adaptive Th1 and Th17 cell response, providing a possible novel therapeutic target for Behcet's disease. The observed discrepancies seem to suggest that the response of CD4^+^T cells to AhR activation may differ among species [30].

In mouse studies it has been reported that the effect of FICZ on Th17 cell differentiation was associated with a reduction in Stat5 phosphorylation [29]. Kimura et al. found that AhR participates in Th17 cell differentiation by regulating Stat1 activation [28]. However, the exact mechanism whereby AhR activation by FICZ or ITE affects CD4^+^T cell polarization is not known in humans. We found that AhR activation of CD4^+^T cells by FICZ or ITE in the presence of antiCD3/CD28 and rIL-23 induced STAT3 and STAT5 phosphorylation, whereas no effect on STAT1 and STAT4 phosphorylation could be detected. It has been shown that Th17 differentiation is inhibited by IL-2 signaling via induction of Stat5 [31] and IL-22 production is associated with STAT3 phosphorylation [32–35]. We hypothesize that in our experiments Stat5 phosphorylation in CD4^+^T cells that were stimulated with FICZ or ITE may lead to an inhibition of IL-17 expression, whereas STAT3 phosphorylation may be associated with the induction of IL-22. Additional studies are needed to support this assumption.

We further showed that the AhR ligands induced a decreased gene expression of T-bet and RORC in CD4^+^T cells. T-bet and RORC are the Th1 and Th17 cell master transcription factors, respectively. The FICZ- or ITE-mediated suppression on Th17 cell polarization was also associated with a decreased gene expression of CCR6 and IL-23R. These findings suggest that AhR activation by FICZ or ITE inhibited not only the development of Th17 cells but also the molecules that are relevant to Th17 cell function and migration.

## 5. Conclusion

Our findings suggest that AhR activation by FICZ or ITE can potentially modulate an aberrant immune response by inhibiting Th1 and Th17 cell responses. Further studies are needed to show whether modulation of the AhR pathways may offer a possible novel therapeutic approach for BD and other autoimmune diseases which are mediated by an aberrant Th1 and Th17 immune response.

## Supplementary Material

Supplementary Figure 1*：*PBMCs from active BD patients (n = 6) and normal controls (n = 6) were stimulated with anti-CD3/CD28 in the presence or absence of FICZ (100 nmol/L) or ITE (100 nmol/L) for 3 days. The cells were analyzed for apoptosis by flow cytometry. Dot plots of a representative subject for each group are shown.
