# Supplementary figures and images for: Decreased Expression of the Aryl Hydrocarbon Receptor in Ocular Behcet's Disease
Source: Mediators Inflamm. 2014 Jun 22;2014:195094. doi: 10.1155/2014/195094 (PMC4090433; doi:10.1155/2014/195094)

**Ctrl**

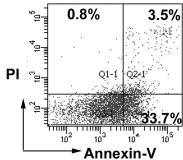

**FICZ**

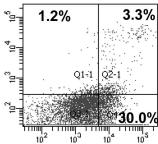

**ITE**

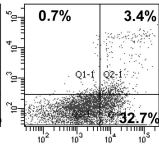

Supplement: Supplementary file 1 — Supplementary Figure 1：PBMCs from active BD patients (n = 6) and normal controls (n = 6) were stimulated with anti-CD3/CD28 in the presence or absence of FICZ (100 nmol/L) or ITE (100 nmol/L) for 3 days. The cells were analyzed for apoptosis by flow cytometry. Dot plots of a representative subject for each group are shown. [file 195094.f1.pdf]
